# Confocal evaluation of lymphangioma circumscriptum^⋆^

**DOI:** 10.1016/j.abd.2022.08.014

**Published:** 2023-09-22

**Authors:** Camila Schlang Cabral da Silveira, Renata Miguel Quirino, Carlos Baptista Barcaui, Luna Azulay-Abulafia

**Affiliations:** Department of Dermatology, Hospital Universitário Pedro Ernesto, Universidade do Estado do Rio de Janeiro, Rio de Janeiro, RJ, Brazil

Dear Editor,

An 18-year-old female student was seen at the Dermatology Clinic, complaining of the progressive increase of lesions on the trunk, without a diagnosis until that moment. Her mother reported the existence of three painless exophytic lesions since birth on the left flank. They progressively increased in size and number, converging and extending to the left side of the trunk. She denied pain or pruritus. On dermatological examination, multiple papules and grouped translucent vesicles measuring between 2 and 9 mm in diameter converged into a large irregular plaque, measuring approximately 20 cm in its longest axis (Fig. 1).

**Figure 1:**
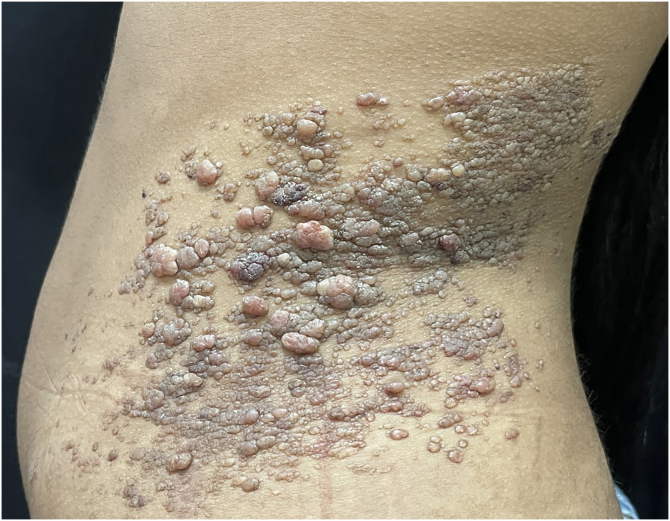
Lymphangioma circumscriptum. Erythematous-brownish papules and translucent vesicles converging into an irregular plaque, approximately 20 cm in size, on the left flank

Dermoscopy revealed pink lacunae with serohematic content, scales on the lesion surface, and pale septa, with a ‘hypopyon‐like’ aspect inside some lesions, orange-brown in the upper portion and red-violet in the lower portion (Fig. 2).

**Figure 2:**
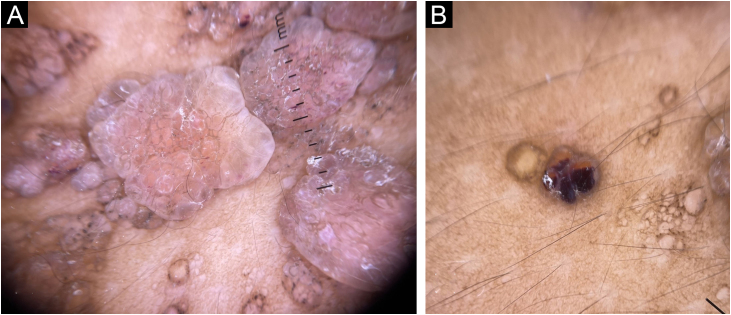
Dermoscopy. (A) Pattern of pink lacunae with pale septa. (B) Lacunar pattern with a “hypopyon‐like” aspect

Confocal microscopy was performed, showing numerous dark cavities in the epidermis and superficial dermis, with high refraction well-defined edges surrounding these cavities and some small shiny dots inside them (Fig. 3).

**Figure 3:**
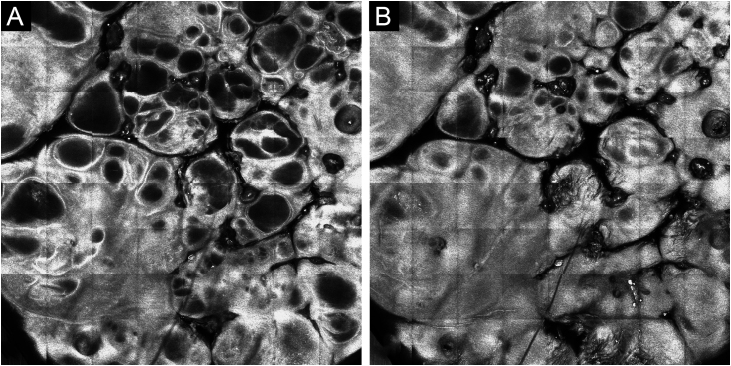
Confocal microscopy. (A) Dark cavities in the superficial dermis, with well-defined edges of high refraction. (B) Dark cavities in the epidermis, with small shiny dots inside

Histopathology showed proliferation of dilated lymphatic vessels lined by a single layer of endothelial cells, confirming the clinical diagnosis (Fig. 4).

**Figure 4:**
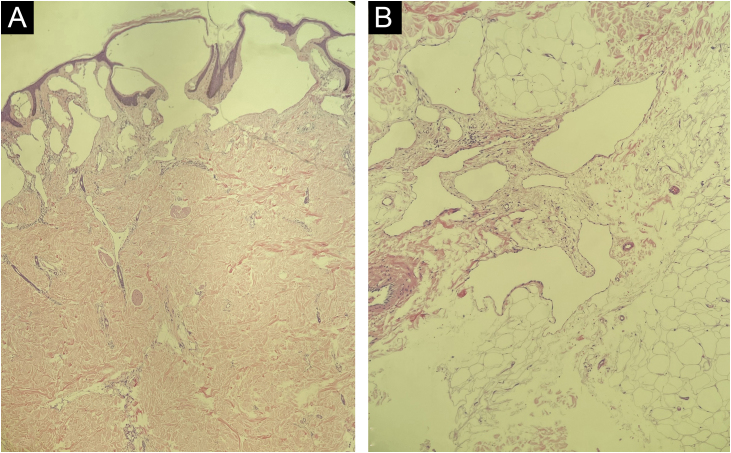
Histopathology. (A) Proliferation of dilated lymphatic vessels in the papillary dermis. (B) Dilated lymphatic vessels in the subcutaneous tissue

Lymphangiomas are rare lymphatic malformations,^1^ which can be congenital or acquired, localized or generalized.^2^ They account for 4% of vascular tumors and 25% of benign vascular tumors in childhood.^2^ The classification of lesions is carried out in two different ways: classification into simple, cavernous and cystic types, with the simple type renamed as lymphangioma circumscriptum (LC),^3^ or classification is divided into superficial, which corresponds to CLC (congenital) and lymphangiectasia (acquired); and deep, corresponding to cavernous lymphangiomas and cystic hygromas.^4^ The patient reported herein corresponds to the most common form of lymphangioma, that is CLC.^1^

CLC is characterized by translucent vesicles, which in half of the cases are present at birth, and when that does not occur, they usually develop before the second year of life, increasing in size and number over the years.^2^ The most affected sites are the proximal areas of the limbs, flank, and perineum, and they may also affect the mucous membranes, including the tongue.^3^ The case described herein corroborates the literature findings, with the appearance of a few lesions since birth, increasing in number over the years, on the flank.

Dermoscopy shows that the predominant pattern in CLC is the lacunar, which helps in the diagnosis.^2^ These lacunar structures are present in 89% of the cases.^5^ The case described in the present report shows the lacunar pattern and is noteworthy the presence of hypopyon-like structures, as these bipolar lacunae are described in only 42% of CLC cases.^5^ Scales, which are also present in the case reported, are found in only 7% of CLC cases.^5^ Other dermoscopy findings that may also be identified are vascular structures and white lines.^5^ The diagnostic definition is complete, with the identification of thin-walled dilated lymphatic vessels on histopathology.^1^, ^2^ Such vessels were present on histopathology in the skin biopsy of the patient reported here. Imaging exams are important to assess the depth and allow the definition of the best therapeutic approach, with magnetic resonance imaging being the method of choice to date, not available in the present case.^2^

*In vivo* reflectance confocal microscopy (RCM) disclosed: 1) numerous dark cavities in the epidermis and upper dermis, which correspond to lacunae; 2) high refraction well-delimited edges around dark spaces, corresponding to thin septa; 3) small shiny structures inside the cavities, which may correspond to lymphoid cells; 4) absence of or very low blood flow.^1^ These findings coincide with what was identified in the confocal microscopy analysis of the patient in the present case, allowing the observation of the structures and correlating them with what was seen on dermoscopy and histopathology.

The treatment of choice for CLC remains surgical excision, but it is unfeasible in very extensive cases, such as this one with 20 cm in its longest axis.^3^

The present article reports a patient with a congenital CLC of progressive growth, which became exuberant, and its findings on confocal microscopy, which are rarely described in the literature and can help in the non-invasive diagnosis.

## Financial support

None declared.

## Authors' contributions

Camila Schlang Cabral da Silveira: Drafting and editing of the manuscript; design and planning of the study; collection, analysis and interpretation of data; intellectual participation in propaedeutic and/or therapeutic conduct of the studied cases; critical review of the literature; approval of the final version of the manuscript.

Renata Miguel Quirino: Drafting and editing of the manuscript; collection, analysis and interpretation of data; intellectual participation in the propaedeutic and/or therapeutic conduct of the studied cases; critical review of the literature; approval of the final version of the manuscript.

Carlos Baptista Barcaui: Drafting and editing of the manuscript; design and planning of the study; collection, analysis, and interpretation of data; intellectual participation in the propaedeutic and/or therapeutic conduct of the studied cases.

Luna Azulay-Abulafia: Drafting and editing of the manuscript; design and planning of the study; collection, analysis, and interpretation of data; intellectual participation in the propaedeutic and/or therapeutic conduct of the studied cases; approval of the final version of the manuscript.

## Conflicts of interest

None declared.
